# A clinical evaluation of amlexanox oral adhesive pellicles in the treatment of recurrent aphthous stomatitis and comparison with amlexanox oral tablets: a randomized, placebo controlled, blinded, multicenter clinical trial

**DOI:** 10.1186/1745-6215-10-30

**Published:** 2009-05-06

**Authors:** Wenxia Meng, Yi Dong, Jie Liu, Zhi Wang, Xiaobo Zhong, Ruiyang Chen, Hongmei Zhou, Mei Lin, Lu Jiang, Feng Gao, Tao Xu, Qianming Chen, Xin Zeng

**Affiliations:** 1 State Key Laboratory of Oral Diseases, West China College of Stomatology, Sichuan University, Sichuan, PR China; 2 Department of Oral Medicine, Stomatology Hospital of Wenchow, Zhejiang, PR China; 3 Section of Oral Biology, College of Dentistry, the Ohio State University, Columbus, Ohio, USA; 4 Hospital of Stomatology, Chonqing University of Medical Sciences, Chongqing, PR China; 5 Hospital of Stomatology, Tianjin, PR China; 6 Statistic Department of Peking Union Medical College, Beijing, PR China

## Abstract

**Background:**

Amlexanox has been developed as a 5 percent topical oral paste for the treatment of patients with recurrent aphthous stomatitis (RAS) in most European countries. However, it is not yet available in China and has not been generally accepted in clinical treatment. The aim of this study was to explore the effectiveness of amlexanox oral adhesive pellicles in the treatment of minor recurrent aphthous ulcers, and compare the results with those of amlexanox oral adhesive tablets in order to analyse the difference between the two dosage forms of amlexanox.

**Methods:**

We performed a randomized, blinded, placebo-controlled, parallel, multicenter clinical study. A total of 216 patients with minor recurrent aphthous ulcers (MiRAU) were recruited and randomized to amlexanox pellicles or placebo pellicles. Pellicles were consecutively applied four times per day, for five days. The size and pain level of ulcers were measured and recorded on treatment days 0, 4 and 6. Finally, the results were compared with those of our previous 104 cases treated with amlexanox tablets.

**Results:**

Amlexanox oral adhesive pellicles significantly reduced ulcer size (P= 0.017 for day 4, P=0.038 for day 6) and alleviated ulcer pain (P=0.021 for day 4, P=0.036 for day 6). No significant difference was observed in the treatment effectiveness between the pellicle and tablet form of amlexanox.

**Conclusions:**

Amlexanox oral adhesive pellicles are as effective and safe as amlexanox oral adhesive tablets in the treatment of MiRAU for this Chinese cohort. However, pellicles seem to be more comfortable to use when compared with the dosage form of tablets. Therefore, in clinical practice, amlexanox oral adhesive pellicles may be a better choice for RAS patients.

**Trials registration:**

Nederlands Trial Register NTR1727.

## Introduction

Recurrent aphthous stomatitis (RAS) is one of the most common oral mucosa diseases. It can affect both men and women of all ages, races and geographic regions. Minor Recurrent aphthous ulceration (MiRAU) is the most common form, which accounts for approximately 70% to 87% of the population with RAS [1,2] and usually has 1 to 5 ulcers at one episode, with each ulcer varying from 3 to 10 mm [3]. For RAS patients, the ulcer pain associated with each episode may severely interfere with eating, speaking, and swallowing.

Although RAS represents a very common oral lesion, its etiology is unknown. Some studies [4,5] have showed that several local and system factors, such as local trauma, immunodeficiency, Haematinic or zinc deficiency and hormonal changes may play a role in the pathogenesis of RAS. Suspected bacteria and viruses may also associate with RAS [6]. Since the etiology is unknown, no curative therapy is available at present. All available systemic or topical treatment methods nowadays are to relieve symptom and accelerate healing. Most systemic medications, although effective, have side effects that limit their general use. Therefore, topical agents remain the first choice for the treatment of RAS, due to their effectiveness and safety.

Amlexanox (C_16_H_14_N_2_O_4_) is a topical anti-inflammatory, anti-allergic drug. It has been developed as a 5% topical oral paste for the treatment of patients with RAS [7-9] and is currently the only clinically proven product approved by the US FDA for the treatment of aphthous ulcers [10]. However, it is not available in China yet. In our previous clinical trial [11], we have demonstrated that amlexanox oral adhesive tablets (active component is 2 mg amlexanox) are effective in the treatment of RAS without major side effects recorded, which were consistent with the previous trials [8,9]. However, during that study, we noticed that some patients complained about the lack of adherence of amlexanox adhesive tablets in some fricative areas and the obvious feeling of extraneous material which made them quite uncomfortable. To solve these problems, another form of amlexanox–oral adhesive pellicles was developed and studied in this randomized, blinded, placebo controlled, parallel, multicentre clinical trial. The amlexanox pellicles contained the same ingredients as the amlexanox tablets, with carrier material being the only difference.

The aims of this study are to evaluate the effectiveness and safety of amlexanox oral adhesive pellicles in the treatment of MiRAU and to compare the results of amlexanox oral adhesive pellicles with our previous data [11] on amlexanox adhesive tablets to analyze the effectiveness, safety, comfort, and convenience of these two amlexanox forms. The study design, assessment criteria and analysis method of our current and previous studies were exactly the same.

## Methods and materials

### Study design

The study was a randomized, blinded, placebo controlled, parallel, multicentre, clinical design. The protocol was reviewed by the Institutional Ethics Committee (IEC)/Institutional Review Board (IRB) of Sichuan University, and each subject signed a detailed informed consent form. All patients were instructed to apply one pellicle to that identified ulcer 4 times a day (after meals and before bedtime) for 5 days (day 1 to day 5). The baseline parameters were taken and recorded on the day of the first visit. Effectiveness and safety evaluations were made on the morning of Day 4 visit and Day 6 visit.

### Blinding

The patients with MiRAU were assigned to treatment group (amlexanox adhesive pellicles) or placebo group by using a computer-generated random number list. The treatment and the placebo agents were packed in identical looking containers and labeled with a code number A or B randomly (named agents A and B) by a statistician from Peking Union Medical College. The code record was kept in Beijing Fu Rei Kang Zheng Medical and Pharmaceutical Institute. Participants assigned to the treatment A received agents A while those assigned to treatment B received agents B. All the investigators/evaluators and patients who participated were blinded to the treatment and placebo agents.

### Methods

The index ulcer's size were measured by using a similar methodology as described before [11] on treatment Days 0, 4 and 6. The investigators measured the maximum and minimum diameters when the ulcer had an oval shape, using a calibrated dental probe with millimeter markings. The two measurements were then multiplied to represent the cross-sectional areas of the ulcer.

To evaluate pain, a visual analog scale (VAS) consisting of a 10-cm horizontal line between poles connoting no pain (origin) to unbearable pain was used. Subjects were told to mark the line with a vertical line at the point that best represented the present pain level of the ulcer.

The effectiveness indics (EI) of the ulcer size and pain improvement were calculated with the following formula (V4 and V6 referring to the values measured at Day 4 visit and Day 6 visit, while V1 referring to the baseline value measured before the study entry): EI = [(V1 – V4 or V6) ÷ V1] × 100%. The effectiveness indices were evaluated on a 4-rank scale: (1) Heal: EI ≥ 95%; (2) Marked improvement: EI<95%, but ≥ 70%; (3) Moderate improvement: EI<70%, but ≥ 30%; (4) No improvement: EI <30%.

All subjects were monitored on the day of the first visit (Day 0) and the end of the study (Day 6) for the occurrence of potential laboratory abnormalities. The evaluations included the following indices: complete blood cell count with differential and platelet count; Alanine Aminotransferase (ALT); Aspartate Aminotransferase (AST); Blood urea nitrogen (BUN); Creatinine (Cr). All the urine and serum tests were performed at the site-specific labs in each center.

Two doctors in Oral Medicine with at least 5 years of expertise from each of the dental faculties were chosen as evaluators in their respective centers, and were especially trained by the main researcher at their institutions to standardize clinical diagnosis and data collection.

### Materials

Amlexanox is 2-amino-7-isopropyl-5-oxo-5H-(1) benzopyrano-(2, 3-b) – pyridine-3- carboxylic acid. Amlexanox adhesive pellicles are lamellar with white color and contain 2 mg amlexanox (identical to the Food and Drug Administration-approved drug in USA for the treatment of canker sores, OraDisc A) with the following ingredients: carboxymethy1 cellulose (CMC), hydroxypropylmethy1 cellulose (HPMC), carbomer, and magnesium stearate. The placebo pellicles contain the above-mentioned excipients except for the active ingredient (amlexanox). The dose and formulation have been approved by the SFDA (State Food and Drug Administration) in China.

### Patient group

All the clinical patients were recruited by advertising in the general population at the study sites or from the clinical patients of the participation centers, with identical inclusion and exclusion criteria (Appendix).

To compare the effectiveness, safety of oral adhesive pellicles with those of another dosage form of amlexanox, the amlexanox oral adhesive tablets, the results of 108 subjects treated with pellicles in this clinical trial were compared with the 104 subjects treated with tablets in our previous study [11]. Furthermore, 21 subjects who had enrolled in both above-mentioned clinical trials were requested to take a questionnaire to subjectively evaluate the comfort and convenience of both dosage forms of amlexanox.

### Statistical analysis

Background and medical history data were summarized with descriptive statistics, and a sample size of 216 subjects were calculated by using an alpha error of 0.05 and beta of 0.1. The *t*-test was performed to compare continuous variables, while the chi-squared test to compare categorical variables. According to the data distribution, Mann-Whitney *U *test was used in the comparison of group differences between the amlexanox and placebo controlled group on Day 4 and Day 6 visit. To evaluate the effectiveness, the Chi-square test and the Mann-Whitney *U *test were used. All data were analyzed by Peking Union Medical College using SPSS software (SPSS 12.0 for Windows; SPSS Inc., Chicago, III). P < 0.05 was considered statistically significant.

## Results

### Demographics

A total of 216 subjects were enrolled in this study, with 140 at center 1 (West China College of Stomatology, Sichuan University), 34 at center 2 (Hospital of Stomatology, Chongqing University of Medical Sciences), and 42 at center 3 (Hospital of Stomatology, Tianjin City). Among all the 216 subjects, 21 subjects also participated our previous study [11]. Only 1 subject of the amlexanox group and 2 subjects of the placebo controlled group were excluded for not following the instructions by the doctor. Therefore, 213 subjects (108 subjects in the amlexanox group and 105 subjects in the placebo controlled group) fulfilled the study. As the discontinuation rate (1.38%) was quite low, we evaluated demographic and effectiveness data without these 3 subjects, which made no influence on any of the interpretation or conclusions.

By the blinded randomization procedures, the amlexanox group and the placebo controlled group were similar as to demography including age, sex, medical history, known allergies, and baseline values of ulcer history, size, and pain (Additional file 1). No abnormal laboratory values were found at study entry between the two groups.

### Amlexanox significantly reduced the size of the ulcers

**Figure 1:**
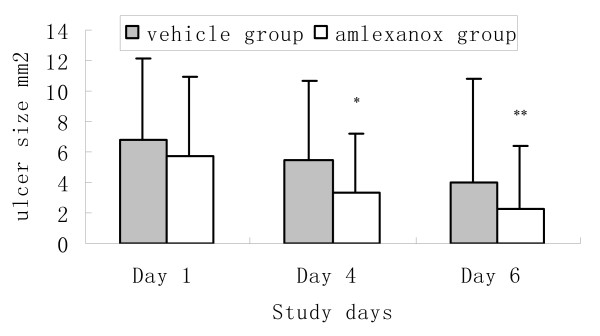
**The comparison of ulcer size between amlexanox group and placebo controlled group during follow-up**. *Mean ulcer size in the amlexanox group was significantly smaller compared with that of the placebo group, Mann-whitney U test. P* = 0.017; P** = 0.038.

Ulcer size between the amlexanox and placebo controlled group was significantly different at the Day 4 and Day 6 visit, respectively (P = 0.017, P = 0.038; Figure 1).

At the Day 4 visit, the effectiveness index of the amlexanox group was much greater than that of the placebo controlled group (P < 0.001). The amlexanox group had a statistically significant higher "improvement" rate (66.67% vs 43.81%, P < 0.001), as well as a significantly higher "marked improvement" rate (41.67% vs 17.14%, P < 0.001) when compared with that of the placebo controlled group (Additional file 2).

At the Day 6 visit, compared with those of the placebo controlled group, the amlexanox group maintained a significantly greater effectiveness index (P < 0.001), the "improvement" rate (86.11% vs 63.81%, P < 0.001), and "marked improvement" rate (73.15% vs 50.48%, P < 0.001; Additional file 2).

### Amlexanox significantly moderated the pain of the ulcers

**Figure 2:**
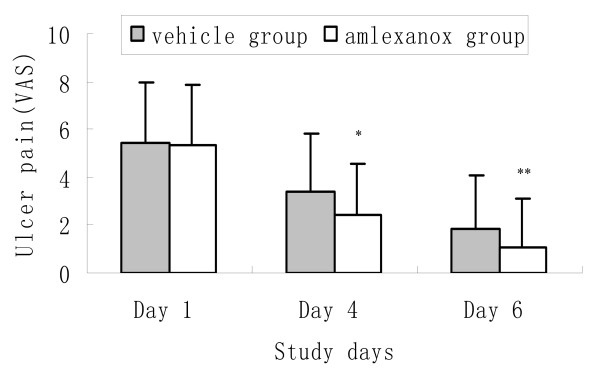
**The comparison of ulcer pain moderation between amlexanox group and placebo controlled group during follow-up reported by all patients'**. *Mean pain score in the amlexanox group was significantly smaller compared with that of the placebo group, Mann-whitney U test. P * = 0.019; P** = 0.03.

Similar results were observed in the ulcer pain resolution between the two groups at the Day 4 and Day 6 visit (P = 0.019, P = 0.036, Figure 2).

At the Day 4 visit, the effectiveness index of the amlexanox group was much greater than that of the placebo controlled group (P = 0.001). The amlexanox group had a significantly higher "improvement" rate (78.70% vs 60.95%, P = 0.005), as well as a significantly higher "marked improvement" rate (41.67% vs 26.67%, P = 0.021) when compared with that of the placebo controlled group.

At the Day 6 visit, compared with those of the placebo controlled group, the amlexanox group still presented with a significantly greater effectiveness index (P < 0.001), the "improvement" rate (91.67% vs 73.33%, P < 0.001), and "marked improvement" rate (75.00% vs 54.29%, P = 0.002; Additional file 3).

### Safety evaluation

None of the patients in the study was observed or reported to have any adverse reactions. None of the hematologic values were considered clinically abnormal. There were no laboratory differences between the 2 groups at baseline or Day 6, and there were no significant changes over time.

### Comparison of amlexanox oral adhesive pellicles and amlexanox adhesive tablets

As the data of the oral adhesive pellicles and amlexanox adhesive tablets were obtained in the same centers, the study design, experimental methods and assessment criteria were the same, and the studies used for the comparison were carried out within a limited time window, we compared the results of the two amlexanox's group by using the historical control method [12-15].

Just as demonstrated in Additional file 4, the amlexanox oral adhesive pellicles had the similar effectiveness in ulcer healing compared with that of the tablets. The "improvement" rate of the two Amlexanox's forms in both ulcer size reduction and ulcer pain moderation showed no statistical significance on the follow-up Day 4 visit (P = 0.625, P = 0.580) and Day 6 visit (P = 0.758, P = 0.467). Moreover, No significant difference was obtained in the "marked improvement" rate in ulcer size reduction (P = 0.962, P = 0.758), as well as in ulcer pain moderation (P = 0.425, P = 0.053) on Day 4 and Day 6 visit, respectively.

The statistical results about the questionnaire demonstrated that amlexanox oral pellicles didn't show much difference in convenience as compared with amlexanox oral adhesive tablets (P = 0.528). However, a significant difference in comfort between both groups was obtained (P = 0.001), with the amlexanox oral pellicles were evidently better than the amlexanox oral adhesive tablets (Additional file 5).

## Discussion

The topical agents have been used for improving discomfort caused by RAS, including glucocorticoids, local analgesics, anti-microbial mouthwash [16] and topical paste such as 5% amlexanox [4]. Amlexanox is an anti-allergic and anti-inflammatory agent which can inhibit the formation and release of histamine and leukotrienes from mast cells, neutrophils, and mononuclear cells, possibly through increasing intracellular cyclic AMP content in inflammatory cells, and a membrane-stabilizing effect or inhibition of calcium influx [17]. The therapeutic effect of 5% amlexanox for RAS has been reported in Caucasian in a series of robust clinical trial programs [10,18].

Our present study has demonstrated that the 5% amlexanox oral adhesive pellicles could not only reduce the ulcer size but also resolve the pain of the patients during the RAS treatment lasting for 5 days in a placebo controlled, randomized, blinded, multicentre clinical trial comprising of 213 subjects. The results showed that 86.11% of subjects had significant improvement in ulcer size reduction (EI = 62.79%) vs. 63.81% of those using the placebo (EI = 6.47%) on Day 6 visit. Data of improvement rate in ulcer pain moderation of amlexanox group were 91.67% (EI = 80.88%) vs. 73.33% (EI = 58.80%) of those in the placebo controlled group on Day 6 visit. During the follow-up, none of the study centers has reported any of systemic side effects. All the results in our present study are consistent with those from the previous trials[8-11], although their dosage forms of amlexanox were different from ours.

Interestingly, during the trial, we noted that the difference in the mean ulcer size reduction on Day 6 visit (P = 0.038) tended to be less significant than that on Day 4 visit (P = 0.017) between the amlexanox group and placebo group, as well as in the ulcer pain moderation (P = 0.036 for Day 6, P = 0.021 for Day 4). These phenomena may be due to the fact that these ulcers can usually resolve without any treatment within 7–10 days based on this disease's natural history, as well as the protective effects of the placebo adhesive pellicle in covering the wound [7,8]. Therefore, it is reasonable to prospect that the pain relief and ulcer size reduction are more effective at the early stage at the onset of RAS for the amlexanox treatment.

In our previous study, we have found that amlexanox oral adhesive tablets were also effective in the treatment of MiRAU in Chinese cohort. However, according to patients' reflection of discomfort about amlexanox tablets, we improved another dosage form of amlexanox, amlexanox oral adhesive pellicles, and also evaluated their effectiveness and safety in ulcer treatment. As a result, the adhesive pellicles not only had the same effectiveness and safety but also had better comfort when compared with tablets.

As the adhesive pellicles are thinner and glutinous, they can adhere to the mucosa surface more easily than the tablets, which limit the likelihood that the drug will be rubbed away or rinsed away with saliva flow. Furthermore, the extraneous material feeling of amlexanox pellicles was gentler compared with the tablets according to the patients' remarks. These may be the reasons that oral adhesive pellicles are more comfortable to use than tablets.

### Limitations

In this study, we have noticed that baseline ulcer size in the placebo-controlled group is larger than amlexanox group as listed in Additional file 1. This may influence the results of comparison between the two groups. However, after controlling for the apparent imbalance between the two groups at baseline there was no change in the overall results. Studies on a larger number of subjects may be needed to further confirm the results.

## Conclusion

Amlexanox oral adhesive pellicles significantly reduced ulcer size (P = 0.017 for Day 4, P = 0.038 for Day 6) and alleviated ulcer pain (P = 0.021 for Day 4, P = 0.036 for Day 6). No significant difference was observed in the treatment effectiveness between the pellicle and tablet form of amlexanox. So amlexanox oral adhesive pellicles is as effective and safe as amlexanox oral adhesive tablets in the treatment of MiRAU for this Chinese cohort. However, it seems to be more comfortable to use pellicles when compared with the dosage form of tablets. Therefore, in clinical practice, amlexanox oral adhesive pellicles may be a better choice for the RAS patients.

## Abbreviations

RAS: recurrent aphthous stomatitis; MiRAU: Minnor recurrent aphthous ulcers; CMC: carboxymethy cellulose; HPMC: hydroxypropylmethy cellulose; VAS: visual analog scale; EI: effectiveness indics; ALT: Alanine Aminotransferase; AST: Aspartate Aminotransferase; BUN: Blood urea nitrogen; Cr: Creatinine.

## Competing interests

None of the authors of the amlexanox oral adhesive pellicles Trial has a financial or any other relation that would pose a conflict of interest.

## Authors' contributions

Each of the authors had substantial contributions either on conception and design or on the drafting of the article and critical revision for this important intellectual content. Specifically, WM and QC have made substantial contributions to conception and design, analysis and interpretation of data. Additionally, WM and YD have involved in drafting the manuscript. ZW, XZh, RC, LJ, FG have been following all the patients during the follow-up clinical visits. XZe, JL, HZ and ML have involved in revising it critically for important content. Finally, TX performed the statistical analysis. All authors read and approved the final manuscript.

## Appendix: selection criteria

Inclusion criteria:

1. Males and females aged 18–60 years old

2. Willingness to participate and sign the informed consent forms

3. Presenting with 1 to 5 aphthous ulcers (less than 72 hours' duration) with a size no greater than 5 mm in diameter

4. An expectation that their ulcers normally take 5 or more days to resolve without treatment

5. Normal sense of pain, without anesthesia or paresthesia

Exclusion criteria:

1. A known history of serious drug hypersensitivities

2. Pregnancy and lactation (Urine hCG-positive)

3. Concurrent clinical conditions that could pose a health risk to the subjects, including serious liver, kidney, and heart dysfunctions

4. A history of an immunologic problem

5. Ulcers as a manifestation of a systemic disease process such as ulcerative colitis, Crohn's disease, Behcet's syndrome, or serious anemia

6. Treatment with systemic steroid or other immunomodulatory agents within 1 month before the study entry

7. Use of nonsteroidal anti-inflamatory drugs or oral antihistamines within 1 month prior to the study entry

8. Treatment of the ulcer with any preparation or medication within 72 hours prior to the study entry

9. Treatment with systemic antibiotics within 2 weeks prior to the study entry

10. Attendance of any other clinical trials within 3 months prior to the study entry

## Supplementary Material

Additional File 1 **Demographic and Ulcer history/baseline values of the studied population**. The amlexanox group and the placebo controlled group were similar as to demography. Click here for file

Additional File 2 **Effectiveness of amlexanox oral pellicles in ulcer size reduction**. The effectiveness index of the amlexanox group was much greater than that of the placebo controlled group. Click here for file

Additional File 3 **Effectiveness of amlexanox oral pellicles in ulcer pain moderation**. The amlexanox group significantly alleviated the ulcer pain compared with the placebo group. Click here for file

Additional File 4 **Comparison of the effectiveness of amlexanox oral pellicles and amlexanox oral adhesive tablets in reducing ulcer size and moderating ulcer pain**. The amlexanox oral adhesive pellicles had the similar effectiveness in ulcer healing compared with that of the tablets. Click here for file

Additional File 5 **Subjective evaluation between amlexanox oral pellicles and adhesive tablets**. A significant difference in comfort between both groups was obtained, with the amlexanox oral pellicles were evidently better than the amlexanox oral adhesive tablets. Click here for file
