# Supplementary material for: A clinical evaluation of amlexanox oral adhesive pellicles in the treatment of recurrent aphthous stomatitis and comparison with amlexanox oral tablets: a randomized, placebo controlled, blinded, multicenter clinical trial
Source: Trials. 2009 May 6;10:30. doi: 10.1186/1745-6215-10-30 (PMC2690593; doi:10.1186/1745-6215-10-30)
Supplement: Additional File 1 — Demographic and Ulcer history/baseline values of the studied population. The amlexanox group and the placebo controlled group were similar as to demography. [file 1745-6215-10-30-S1.doc]

**Table 1 Demographic and Ulcer history/baseline values of the studied population**

|  | *Amlexanox group(n=108)* | *Placebo controlled group(n=105)* |  |
| --- | --- | --- | --- |
|  | *n Mean(SD)/proportion* | *n Mean(SD)/proportion* | *P-value* |
| Age (years)  Gender  Women  Men  Weight (kg)  Drug hypersensitivity  Yes  No  Comitant systemic disease  Yes  No  Course (days)  Ulcer history ( years) | 108 29.82 (10.44)    64  44  108 58.83 (10.41)  103 95.4%  5 4.6%  1 0.9 %  107 99.1 %  108 2.65 (0.54)  108 7.45 (5.76) | 105 30.18 (11.06)  71  34  105 58.10 (9.06)  100 95.2 %  5 4.8 %  2 1.9 %  103 98.1 %  105 2.55 (0.62)  105 6.88 (5.66) | 0.809^a^  0.693 ^b^  0.500 ^a^  0.733 ^b^  0.987 ^b^  0.204 ^a^  0.209 ^c^ |
| Anticipated healing time (days) | 108 9.82 (4.53) | 105 9.21 (3.19) | 0.572^c^ |
| Size, mm^2^ | 108 5.68 (5.21) | 105 6.71 (5.39) | 0.096 ^c^ |
| Pain, VAS | 108 5.31 (2.51) | 105 5.42 (2.51) | 0.675 ^c^ |

^a^*t*-test; ^b^chi-square test; ^c^Mann-whitney U test

*VAS*, Visual analog scale
