# Supplementary material for: A clinical evaluation of amlexanox oral adhesive pellicles in the treatment of recurrent aphthous stomatitis and comparison with amlexanox oral tablets: a randomized, placebo controlled, blinded, multicenter clinical trial
Source: Trials. 2009 May 6;10:30. doi: 10.1186/1745-6215-10-30 (PMC2690593; doi:10.1186/1745-6215-10-30)
Supplement: Additional File 2 — Effectiveness of amlexanox oral pellicles in ulcer size reduction. The effectiveness index of the amlexanox group was much greater than that of the placebo controlled group. [file 1745-6215-10-30-S2.doc]

**Table 2** Effectiveness of amlexanox oral pellicles in ulcer size reduction

|  | Day 4 visit | | |  | | Day 6 visit | | | |
| --- | --- | --- | --- | --- | --- | --- | --- | --- | --- |
|  | *Amlexanox*  *group*  *(n=108)* | *Placebo controlled*  *group*  *(n=105)* | *P value* |  |  | *Amlexanox*  *group*  *(n=108)* | | *Placebo controlled*  *group*  *(n=105)* | *P value* |
| (1) Heal  (2) Marked improvement  (3) Moderate improvement  (4) No improvement | 17  28  27  36 | 3  15  28  59 | 56  23  14  15 | | | | 30  23  14  38 | |  |
| Marked improvement rate  (1) + (2)  Improvement rate  (1) + (2) +(3)  Effectiveness index (EI) | 41.67%  66.67% | 17.14%  43.81% | <0.001^*^  <0.001^*^  <0.001^**^ | | 73.15%  86.11% | | 50.48%  63.81% | | <0.001^*^  <0.001^*^  <0.001^**^ |
|  | 38.57% | -4.28% |  |  | 62.79% | | 6.47% | |  |

^*^Chi-square test.

^**^Mann-Whitney *U* test.
