# Supplementary material for: A clinical evaluation of amlexanox oral adhesive pellicles in the treatment of recurrent aphthous stomatitis and comparison with amlexanox oral tablets: a randomized, placebo controlled, blinded, multicenter clinical trial
Source: Trials. 2009 May 6;10:30. doi: 10.1186/1745-6215-10-30 (PMC2690593; doi:10.1186/1745-6215-10-30)
Supplement: Additional File 3 — Effectiveness of amlexanox oral pellicles in ulcer pain moderation. The amlexanox group significantly alleviated the ulcer pain compared with the placebo group. [file 1745-6215-10-30-S3.doc]

**Table 3** Effectiveness of amlexanox oral pellicles in ulcer pain moderation

|  | Day 4 visit | | | |  | Day 6 visit | | |
| --- | --- | --- | --- | --- | --- | --- | --- | --- |
|  | *Amlexanox*  *group*  *(n=108)* | *Placebo controlled*  *group*  *(n=105)* | *P value* | |  | *Amlexanox*  *group*  *(n=108)* | *Placebo controlled*  *group*  *(n=105)* | *P value* |
| (1) Heal  (2) Marked improvement  (3) Moderate improvement  (4) No improvement | 27  18  40  23 | 13  15  36  41 | 71  10  18  9 | | | | 48  9  20  28 |  |
| Marked improvement rate  (1) + (2)  Improvement rate  (1) + (2) +(3)  Effectiveness index (EI) | 41.67%  78.70% | 26.67%  60.95% | 0.021^*^  0.005^*^  0.001^**^ | 75.00%  91.67% | | | 54.29%  73.33% | 0.002^*^  <0.001^*^  <0.001^**^ |
|  | 55.46% | 27.72% |  | 80.88% | | | 58.80% |  |

^*^Chi-square test.

^**^Mann-Whitney *U* test.
