# Supplementary material for: A clinical evaluation of amlexanox oral adhesive pellicles in the treatment of recurrent aphthous stomatitis and comparison with amlexanox oral tablets: a randomized, placebo controlled, blinded, multicenter clinical trial
Source: Trials. 2009 May 6;10:30. doi: 10.1186/1745-6215-10-30 (PMC2690593; doi:10.1186/1745-6215-10-30)
Supplement: Additional File 4 — Comparison of the effectiveness of amlexanox oral pellicles and amlexanox oral adhesive tablets in reducing ulcer size and moderating ulcer pain. The amlexanox oral adhesive pellicles had the similar effectiveness in ulcer healing compared with that of the tablets. [file 1745-6215-10-30-S4.doc]

**Table 4** Comparison of the effectiveness of amlexanox oral pellicles and amlexanox oral adhesive tablets in reducing ulcer

size and moderating ulcer pain

|  | Ulcer size reduction | | |  | Ulcer pain moderation | | |
| --- | --- | --- | --- | --- | --- | --- | --- |
|  | *Pellicles group*  *(n=108)* | *Tablets group*  *(n=104)* | *P value* |  | *Pellicles group*  *(n=108)* | *Tablets group*  *(n=104)* | *P value* |
| **Day 4 visit**  (1) Heal  (2) Marked improvement  (3) Moderate improvement  (4) No improvement  Marked improvement rate  Improvement rate | 17  28  27  36  41.67%  66.67% | 21  22  23  38  41.35%  63.46% | 27  18  40  23  0.962 41.67%  0.625 78.70% | | | 35  14  36  19  47.12%  81.73% | 0.425  0.580 |
| **Day 6 visit**  (1) Heal  (2) Marked improvement  (3) Moderate improvement  (4) No improvement  Marked improvement rate  Improvement rate | 56  23  14 | 62  16  10 | 0.758  0.758 | 71  10  18 | | 82  7  9 | 0.053  0.467 |
|  | 15  73.15%  86.11% | 16  75.00%  84.62% |  | 9  75.00%  91.67% | | 6  85.58%  94.23% |  |

All the statistic assays was done by using the Chi-square test.

Marked improvement rate = (1) + (2), Improvement rate=(1) + (2) + (3)
