# Supplementary material for: A clinical evaluation of amlexanox oral adhesive pellicles in the treatment of recurrent aphthous stomatitis and comparison with amlexanox oral tablets: a randomized, placebo controlled, blinded, multicenter clinical trial
Source: Trials. 2009 May 6;10:30. doi: 10.1186/1745-6215-10-30 (PMC2690593; doi:10.1186/1745-6215-10-30)
Supplement: Additional File 5 — Subjective evaluation between amlexanox oral pellicles and adhesive tablets. A significant difference in comfort between both groups was obtained, with the amlexanox oral pellicles were evidently better than the amlexanox oral adhesive tablets. [file 1745-6215-10-30-S5.doc]

**Table 5** Subjective evaluation between amlexanox oral pellicles and adhesive tablets

|  | Convenience degree *(patients)* | |  | Comfort degree ( *patients)* | |  |
| --- | --- | --- | --- | --- | --- | --- |
|  | Total Good Moderate Bad  Score item ( 3 ) ( 2 ) ( 1 ) | |  | Total Good Moderate Bad  Score item ( 3 ) ( 2 ) ( 1 ) | |  |
| Pellicles | 47 | 11 4 6 |  | 63 21 0 0 |  | |
| Tablets | 48 | 8 6 7 |  | 38 5 7 9 |  | |
| P | 0.464^*^ |  |  | 0.001 ^**^ |  | |

All the statistic assays was done by using the Wilcoxon rank test.

^*^ P >0.05, ^**^ P <0.01
